# Automatic identification and annotation of MYB gene family members in plants

**DOI:** 10.1186/s12864-022-08452-5

**Published:** 2022-03-19

**Authors:** Boas Pucker

**Affiliations:** Institute of Plant Biology & Braunschweig Integrated Centre of Systems Biology (BRICS), Braunschweig, Braunschweig, TU Germany

**Keywords:** MYBs, Croton tiglium, Castanea crenata, Genome-wide, R2R3 MYBs, Orthology, Phylogeny

## Abstract

**Background:**

MYBs are among the largest transcription factor families in plants. Consequently, members of this family are involved in a plethora of processes including development and specialized metabolism. The MYB families of many plant species were investigated in the last two decades since the first investigation looked at *Arabidopsis thaliana*. This body of knowledge and characterized sequences provide the basis for the identification, classification, and functional annotation of candidate sequences in new genome and transcriptome assemblies.

**Results:**

A pipeline for the automatic identification and functional annotation of MYBs in a given sequence data set was implemented in Python. MYB candidates are identified, screened for the presence of a MYB domain and other motifs, and finally placed in a phylogenetic context with well characterized sequences. In addition to technical benchmarking based on existing annotation, the transcriptome assembly of *Croton tiglium* and the annotated genome sequence of *Castanea crenata* were screened for MYBs. Results of both analyses are presented in this study to illustrate the potential of this application. The analysis of one species takes only a few minutes depending on the number of predicted sequences and the size of the MYB gene family. This pipeline, the required bait sequences, and reference sequences for a classification are freely available on github: https://github.com/bpucker/MYB_annotator.

**Conclusions:**

This automatic annotation of the MYB gene family in novel assemblies makes genome-wide investigations consistent and paves the way for comparative studies in the future. Candidate genes for in-depth analyses are presented based on their orthology to previously characterized sequences which allows the functional annotation of the newly identified MYBs with high confidence. The identification of orthologs can also be harnessed to detect duplication and deletion events.

**Supplementary Information:**

The online version contains supplementary material available at 10.1186/s12864-022-08452-5.

## Introduction

MYB transcription factors are named after an avian myeloblastosis virus protein (v-Myb) which is a modified version of the cellular c-Myb and causes the activation of oncogenes [1]. While MYBs were first discovered in animals, they appear in substantially larger numbers in plants and form one of the largest transcription factor families [2–6]. A characteristic MYB feature is the presence of a conserved DNA-binding domain at the N-terminus [7]. Up to four imperfect amino acid repeats (50–53 amino acids) form three alpha-helices each [7]. Helix two and three of each repeat are arranged to form a helix-turn-helix structure [8]. Three regularly spaced tryptophan or other hydrophobic amino acid residues form the core of this structure [8]. The third alpha helix is responsible for the direct DNA interaction [9]. The repeats are classified into R1, R2, and R3 based on similarity to the respective repeats of the first characterized MYB, c-Myb [1]. MYB proteins are classified based on the presence of these repeats. For example, R2R3-MYBs harbor the R2 and R3 repeat while 3R-MYBs have one copy of each of the repeats (R1R2R3). Further classification into subgroups can be achieved based on the phylogenetic relationships and characteristic sequence motifs in the C-terminal region [2, 5, 10]. Different MYB classification systems were proposed in previous studies [2, 5, 10].

R1R2R3-MYBs have been proposed to be regulators of the cell cycle with conserved functions between animals and plants [11, 12]. R2R3-MYBs account for the large MYB family size in plants [2]. The evolutionary origin of R2R3-MYBs and 3R-MYBs is still debated. The loss model proposes that R2R3-MYBs diverged from 3R-MYBs through loss of the R1 repeat [13–15], while the gain model proposes that the 3R-MYBs evolved from the R2R3-MYB through duplication of a repeat [10, 16]. R2R3-MYBs are involved in the regulation of numerous processes including the regulation of developmental processes, response to environmental stresses, and specialized metabolism [17–19]. WEREWOLF/MYB66 is a negative regulator of the root hair formation that determines the pattern of root hairs on the root epidermis of *Arabidopsis thaliana* [17]. An investigation of this MYB based on its crystal structure revealed the DNA binding site AACNGC and also suggests that this MYB is able to differentiate between DNA methylation states [20]. DEFECTIVE IN TAPETAL DEVELOPMENT AND FUNCTION/MYB35 determines the sex of *Asparagus officinalis*, but not in all other species of this genus [21]. The *A. thaliana* PFG1/MYB12 and the paralogs PFG2/MYB11 and PFG3/MYB111 are responsible for the regulation of the flavonol biosynthesis in most tissues [18]. This process appears highly conserved as orthologs of the AtMYB11/AtMYB12/AtMYB111 clade in *Beta vulgaris* [22] and *Medicago truncatula* [19] are also regulators of the flavonol biosynthesis. MYB21 and MYB24 were identified as additional flavonol regulators in the stamen of *A. thaliana* [23]. Some processes like the regulation of the flavonol biosynthesis depend only on MYB regulators [18]. Other specialized metabolite biosynthesis pathways are regulated by the interaction of multiple proteins. The MBW complex, named after the three components MYB, bHLH, and WD40, is one of the best studied transcriptional regulation systems [24–27]. Two branches of the flavonoid biosynthesis, the anthocyanin and proanthocyanidin biosynthesis, are controlled by the MBW complex [24, 25, 28]. Since anthocyanins are responsible for the pigmentation of flowers and other plant structures, mutants in their regulation can be identified based on a visible phenotype. Proanthocyanidins are responsible for the coloration of seed coats thus mutants in their biosynthesis can be identified based on a yellow seed color. Mutations in the regulating MYBs and other transcription factors [29–32] are often the reason for loss of anthocyanins and/or proanthocyanins. Transcriptional regulation was studied based on these pathways due to their visually detectable phenotypes.

Since MYBs are controlling many processes in plants, there is also a substantial interest to understand their functions in crop species. In Brassicaceae, the glucosinolate content controlled by several MYBs is an economically relevant trait [33]. ATR1/MYB34, HIG1/MYB51, and MYB122 increase the indolic glucosinolates and HAG1/MYB28, PMG2/RAO7/MYB29, and MYB76 the aliphatic glucosinolates in *Arabidopsis thaliana* [34–36]. The red coloration of sugar beets is controlled by BvMYB1 which activates two betalain biosynthesis genes [37]. *AmMYB1* in amaranth was identified as best candidate gene to explain the seed coloration variation between accessions [38]. A MYB appears to be the underlying factor of post-harvest hardening that renders a specific yam accession inedible within a day [39, 40]. MYB duplications between different apple cultivars appear responsible for differences in the red fruit flesh coloration [41]. Consumers prefer apricots with a red blush which is controlled by an anthocyanin biosynthesis activating MYB [42]. The identification of MYB candidate genes and the regulated processes is the first step towards modification through SMART breeding or genome editing [43, 44]. This interest in MYBs sparked numerous genome-wide investigations in species with a new genome or transcriptome assembly [3, 4, 22, 31, 45–48]. The identification of MYBs is repeatedly performed on many different data sets with strong variation in the quality of the analyses. Well described *A. thaliana* MYB sequences [2] are often used as baits to find new MYBs based on sequence similarity. Like all routine tasks with clearly defined steps, the identification of MYBs is a promising target for an automatic approach. We previously developed an automatic workflow, called KIPEs, for the annotation of core flavonoid biosynthesis genes which could also be expanded to the annotation of transcription factor gene families [4]. However, KIPEs is optimized for the identification and assessment of enzymes based on conserved amino acids in the active center. One underlying assumption is a small number of gene copies per species, which is violated by the very large MYB gene family. Also it is technically possible to run KIPEs for the identification of a gene family, the performance decreases with gene family size. Many previous studies relied only on BLAST or added additional filters for the presence of conserved R2R3-MYB domains in candidate sequences [4, 22, 49]. The inspection of MYB domains is laborious when performed manually, but suitable to define a set of fully functional R2R3-MYBs. While specificity of this filtering approach is high, it suffers from a low sensitivity i.e. neglects degenerated copies which might have experienced neofunctionalization. There are other solutions to identify orthologous sequences in a large number of species independent of the presence of specific sequence patterns [50, 51], but these approaches would require a substantial amount of manual cleaning to narrow down a final set of MYB sequences. Particular challenges are analyses based on transcriptome assemblies, because transcriptome assemblies show often a large number of isoforms resulting from alternative splicing or artifacts [52, 53].

This study presents a Python-based pipeline to provide a high quality annotation of all MYBs in a given set of peptide or coding sequences that are provided as input. Additionally, MYB candidates are checked for conserved domains and assigned to orthologs in other plant species. Genome sequencing and the construction of assemblies is becoming a routine task. The generation of high quality structural annotations is also advancing quickly with the aid of massive RNA-Seq data sets and full length transcript sequencing. Therefore, a huge number of data sets will be available to study MYBs in an unprecedented number of different plant species. Our automatic identification of MYBs in a large number of species facilitates pan-MYB analyses to better understand the evolution of the MYBome and to transfer functional insights acquired in one species effectively to orthologs in other species.

### Implementation

#### MYB data sets

The identification of MYBs in novel genome or transcriptome sequences requires broad phylogenetic coverage of bait sequences. The MYB domain sequences of *Arabidopsis thaliana* [2], *Vitis vinifera* [3], *Beta vulgaris* [22], *Musa acuminata* [4], *Medicago truncatula*, *Populus trichocarpa*, *Citrus sinensis*, *Solanum lycopersicum*, *Solanum tuberosum*, *Aquilegia coerulea*, *Oryza sativa*, *Zea mays* [47], *Amborella trichopoda*, *Picea abies*, *Selaginella moellendorfii*, *Physcomitrella patens*, *Chlamydomonas reinhardtii*, *Volvox carteri*, *Micromonas pusilla*, *Ostreococcus lucimarinus*, and *Cyanidioschyzon merolae* [10] were merged to generate a bait sequence collection. Closely related non-MYB sequences including CDC5 were identified by running a BLASTp search with the bait MYB sequences against the Araport11 peptide sequences [54]. Hits with a minimum BLAST score of 100 were collected and stripped of any *bona fide* MYBs. This step allowed the identification of MYB-like sequences, but excludes spurious hits that would slow down the following analysis steps. While the previously described MYBs form a collection of 1889 ingroup sequences, these 26 non-MYB sequences represent the outgroup sequences for down-stream analyses.

### Pipeline

**Fig. 1:**
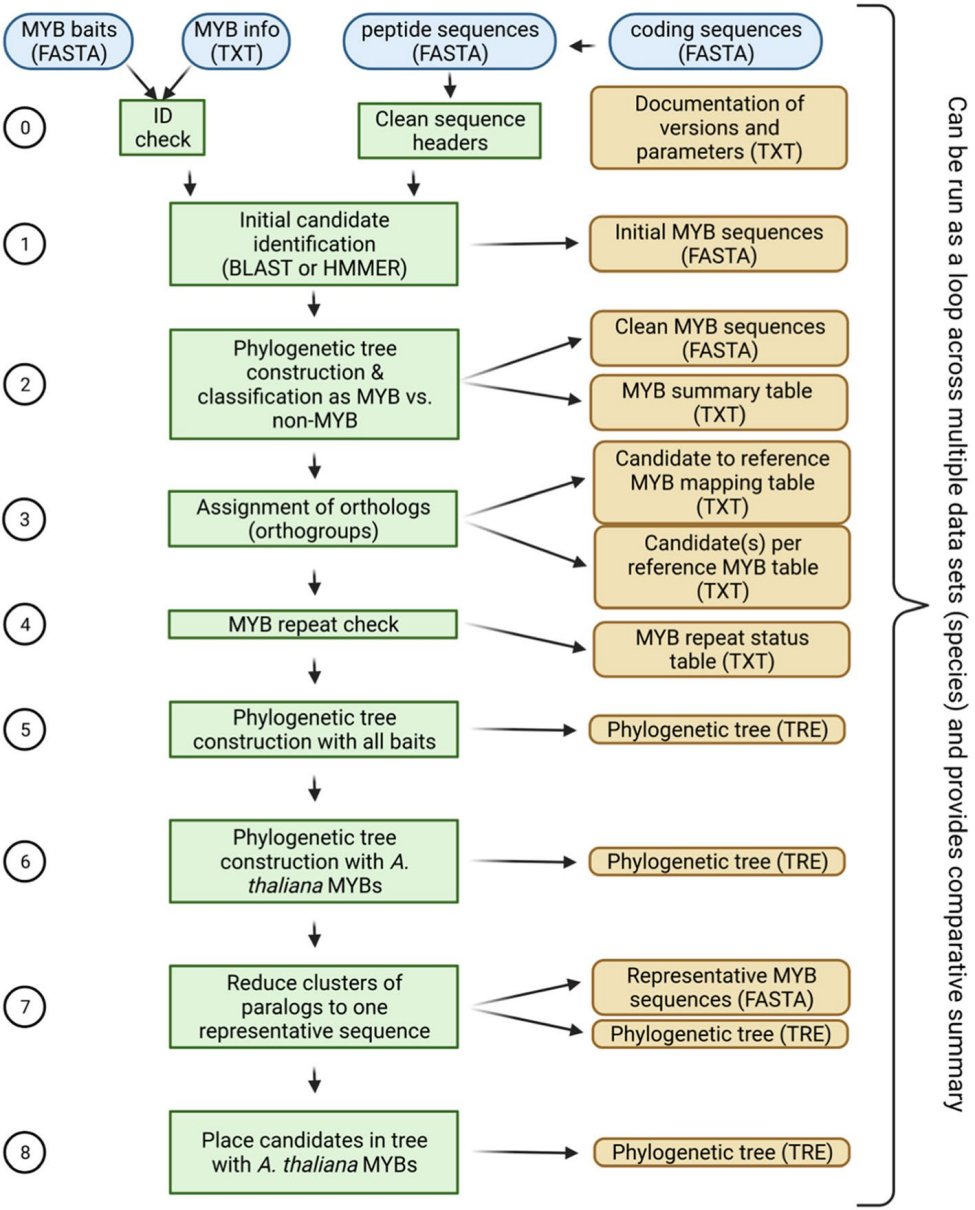
Simplified illustration of a pipeline for the automatic annotation of MYBs. Please refer to the text and the documentation on github for additional details about the pipeline. There is an option to run this pipeline across all provided input files in a folder. This enables the generation of summary files that compare the MYB gene families between the analyzed species

The automatic annotation pipeline is summarized in Fig. 1. Required inputs are (1) the MYB bait sequences (described above), (2) a classification of the MYB bait sequences into ingroup and outgroup, and (3) a set of coding sequences or peptide sequences that will be analyzed. Step 0: All parameters, tool versions, and the input files are logged in a report file for reproducibility. If the Python module hashlib is available, md5sums are calculated for all input files to ensure an accurate documentation. However, calculation of this checksum is optional and file names (including their paths) will be documented in any case. Cleaning of the input sequences removes any characters from the sequence names that would interfere with the phylogenetic analysis. Step 1: Initial candidates are identified based on local sequence similarity via BLAST [55, 56] or HMMER [57]. Default parameters accept BLASTp hits with 50 amino acid length, 80% alignment similarity, and a maximum of 100 hits per bait sequence. This is a very sensitive setting given the large number of bait sequences and an expected MYB gene family size below 300 in most species. If a collection of coding sequences is provided, these will be translated and then compared based on BLASTp to harness the stronger conservation of amino acid sequences compared to nucleotide sequences. Step 3: A phylogenetic tree is constructed with these initial candidates and all bait sequences. Alignments are constructed with MAFFT [58]. Tree construction via RAxML [59] and FastTree2 [60] is supported. FastTree2 (-wag -nopr -nosupport) is recommended due to substantially higher speed when large data sets are analyzed. Step 3: Calculation of distances between different leaves of the tree is used to identify ortholog relationships between bait and candidate sequences. The Python package dendropy [61] is applied using the patristic distance method and counting edges. To exclude outliers caused by fragmented sequences or annotation artifacts, candidates are excluded if the distance to the next bait sequences exceeds three times the average distance of nearest neighbours. This cutoff was optimized by manually inspecting distributions of this value in context with the corresponding phylogenetic trees, but it is possible to modify this value as well as most other parameters. The bait sequences with the shortest distance are identified for each selected candidate in the tree. If most of these bait sequences are ingroup MYBs, the candidate is classified as MYB (Additional file 1). If most of these bait sequences are outgroup sequences, the candidate is classified as a MYB-like sequence (aka non-MYB). All sequences passing this filter are considered clean candidates. Step 4: A check for the presence of MYB repeats is performed based on regular expressions (see documentation for details) derived from previously reported alignments [10, 62]. A repeat-based MYB classification is widely used and also supported here. However, it is important to note that these groups do not represent monophyletic groups. Step 5: A new phylogenetic tree is constructed with the clean MYB candidates and all bait sequences. Step 6: An optional step assigns all newly discovered MYBs to a group of reference MYBs e.g. the well characterized *A. thaliana* MYBs. Based on the assumptions that orthologs are likely to have the same functions, this generates hypotheses about the function of the newly discovered MYBs. Additionally, it is possible to identify the expansion and contraction of specific MYB lineages compared to this reference. Step 7: It is possible to collapse large groups of very similar sequences in the analyzed data set and to represent these by only the longest sequence. This option is intended for transcriptome assemblies which can include large numbers of isoforms caused by alternative splicing and artifacts. Step 8: A new phylogentic tree of the representative sequences identified in step 7 and the reference sequence set (e.g. *A. thaliana* MYBs) is constructed.

## Results and discussion

### Proof of concept and benchmarking

Several benchmarking data sets were analyzed to ensure that the pipeline performs well for a range of different plant species. Araport11 sequences of the *A. thaliana* accession Col-0 [54] were analyzed and the well characterized MYBs were recovered (example output files on github). This demonstrates that the pipeline works as expected. The annotated sequences of the *A. thaliana* accession Nd-1 [63] were screened for MYBs. As expected, there is a 1:1 relationship between the MYBs of Col-0 and Nd-1 (Additional file 2). This demonstrates that not just identical, but also slightly different sequences are accurately identified.

### Performance

**Fig. 2:**
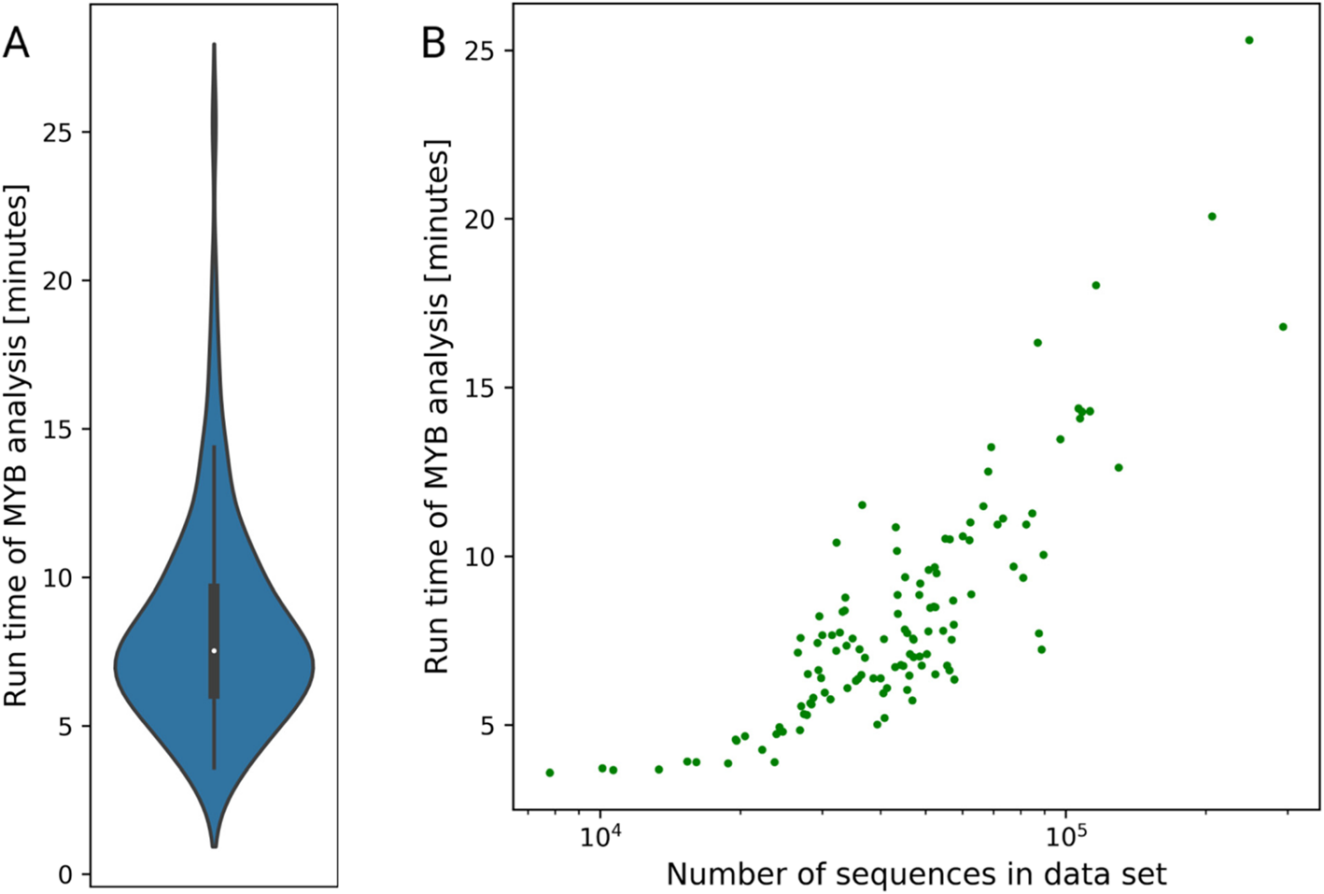
Average run time per data set (species) with default parameters based on coding sequences (**A**). Positive correlation of run time with the number of sequences in the data set (**B**)

Benchmarking and performance tests were performed on a compute cluster without control over other jobs running on the same machine. This prevented a precise and informative calculation of run times, but also represents realistic conditions. A total of 121 coding sequence sets were downloaded from Phytozome [64] and screened for MYBs. The average run time per species using default parameters, 4 CPUs, FastTree, and v0.153 of the pipeline was about 8 min (Fig. 2, Additional file 3). The memory requirements of all steps in the pipeline are very low (< 1 GB). The major factor contributing to the run time is the construction of a phylogenetic tree. However, the use of RAxML takes substantially longer. If a job is canceled, the analysis can continue at the last completed check point or at the last analyzed data set (species), respectively. The required hard disk space is minimal (63.5 MB for *A. thaliana*). Changes in the parameters and especially in the number of supplied bait sequences can alter the computational costs substantially. While there are differences with respect to the run time depending on the number of predicted sequences per species and the size of the MYB gene family, this analysis indicates that large data sets can be processed effectively.

### Discovery of MYBs in the *Castanea crenata* genome sequence

**Fig. 3:**
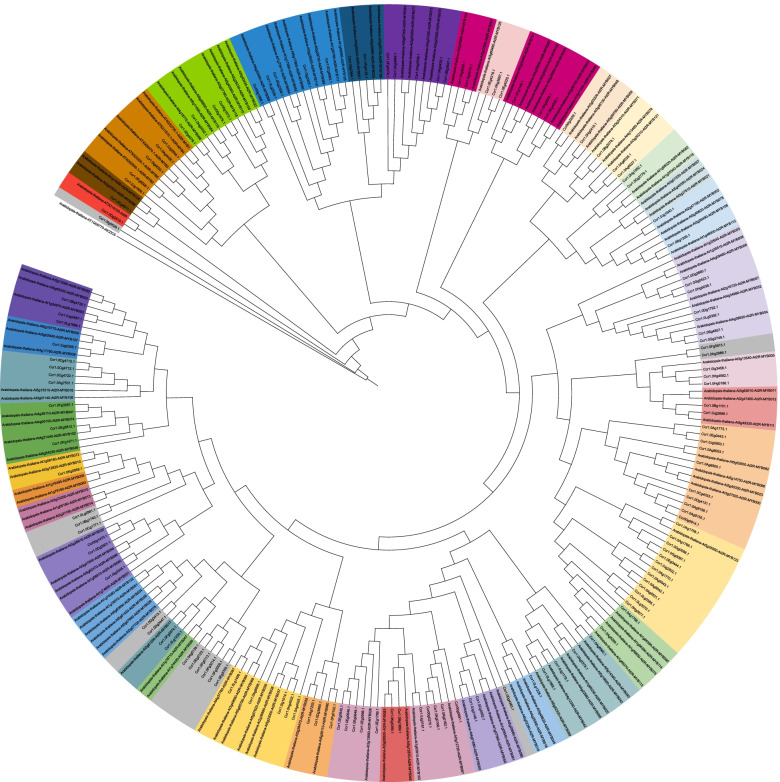
Relationships of *Castanea crenata* MYB candidates and well characterized *Arabidopsis thaliana* MYBs. This figure was constructed with iTOL [69]

As a proof of concept, MYBs encoded in the recently sequenced *Castanea crenata* genome [65] were investigated. The predicted peptide sequences were screened with default parameters and resulted in the identification of 136 MYBs (Fig. 3, Additional file 4). A R2R3-MYB domain was detected in 112 of them. No orthologs of the *Cruciferae*-specific glucosinolate biosynthesis regulating MYBs AtMYB028, AtMYB029, AtMYB034, AtMYB051, AtMYB076, and AtMYB122 were detected in *C. crenata*. This is not surprising, because *C. crenata* belongs to the *Fagaceae,* and also in line with previous reports about the absence of this MYB lineage from non-Cruciferae [3]. Regulators of the flavonoid biosynthesis (AtMYB011/AtMYB012/AtMYB111, Ccr1.0Bg1101.1-S7/Ccr1.0Jg2696.1-S7), anthocyanin biosynthesis (AtMYB075/AtMYB090/AtMYB113/AtMYB114, Ccr1.0Ag5288.1-S6), and proanthocyanidin biosynthesis (AtMYB123, Ccr1.0Ag1758.1 / Ccr1.0Ag1766.1 / Ccr1.0Ag1768.1 / Ccr1.0Ag1770.1 / Ccr1.0Ag1773.1 / Ccr1.0Ag5531.1 / Ccr1.0Ag5542.1 / Ccr1.0Ag5543.1 / Ccr1.0Eg0443.1 / Ccr1.0Eg0444.1 / Ccr1.0Gg0097.1 / Ccr1.0Gg0098.1 / Ccr1.0Hg2677.1 / Ccr1.0Jg0953.1 / Ccr1.0Jg2532.1 / Ccr1.0Lg0385.1 / Ccr1.0Lg3370.1) were detected. In general, copy number differences can be explained by lineage-specific duplication events. Therefore, it is not possible to establish 1:1 relationships between *A. thaliana* and *C. crenata*. Interestingly, there are multiple homologs of AtMYB123 in *C. crenata* which could indicate a high importance of proanthocyanidins in this species. Further investigations could analyze the transcription of these genes to exclude unexpressed copies. The reduced number of PAP homologs could suggest a lower importance of anthoycanins. While the copies of the anthocyanin regulator show different functions in *A. thaliana* [66], loss of MYB114 in several *A. thaliana* accessions including Col-0 [67] and Nd-1 [68] suggest that there is functional redundancy between them.

### Discovery of MYBs in the *Croton tiglium* transcriptome assembly

**Fig. 4:**
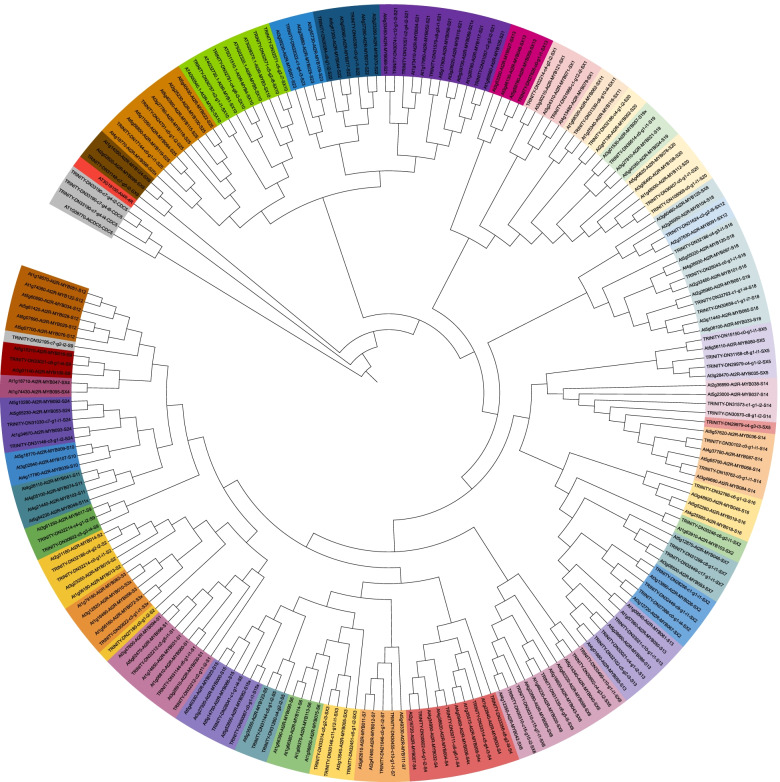
Relationships of *Croton tiglium* MYBs and well characterized *Arabidopsis thaliana* MYBs. This figure was constructed with iTOL [69]

To demonstrate that the pipeline also works for inherently incomplete transcriptome assemblies, MYBs were investigated in the transcriptome assembly of *Croton tiglium* [70]. An analysis with default parameters revealed 140 MYBs (Fig. 4, Additional file 5). This includes 103 candidates with a complete R2R3-MYB domain. Transcriptome assemblies are known to be rich in isoforms of the same genes, which can be due to alternative splicing or artifacts. Clusters of these isoforms were represented by the longest sequence among them. This reduced the number of MYB candidates to 79 including 69 with a R2R3 domain. Not all *A. thaliana* MYBs are matched by orthologs in *C. tiglium*. Although this transcriptome assembly is based on paired-end RNA-seq data sets representing leaf, root, stem, seed, and inflorescence, some not or lowly expressed MYBs might not be represented in the assembly. Therefore, they cannot be identified in this analysis. Again, the absence of orthologs of the glucosinolate regulating MYBs aligns well with previous reports [3], because *C. tiglium* belongs to the *Euphorbiaceae*. The conserved regulators of the flavonol biosynthesis (AtMYB011/AtMYB012/AtMYB111, TRINITY-DN21046-c0-g1-i2-S7) and proanthocyanidin biosynthesis (AtMYB123, TRINITY-DN31260-c4-g2-i2-S5) were detected. These findings are in line with previous reports of the flavonol regulators and proanthocyanidin regulator being detectable in this transcriptome assembly [71]. The absence of a PAP ortholog from the assembly is not surprising, because none of the sampled tissues showed a pigmentation by anthocyanins [70]. Anthocyanin regulators are well known to be lowly expressed in tissues without anthocyanin pigmentation [72, 73] thus a lack of expression is a likely explanation of this result.

### Limitations and next steps

The collection of bait sequences distributed with the tool should be appropriate for most applications. This collection covers a large taxonomic range including chlorophytes, charophycean algae, bryophytes, and vascular plants. The dominance of sequences belonging to vascular plants can be explained by the generally larger MYB families in these species. The major MYB lineages are represented in this collection, but MYB lineages that are restricted to certain taxonomic groups could be missing. While the initial identification based on overall sequence similarity is a robust approach, the precise classification and functional annotation of such MYBs could be less accurate due to the lack of close orthologs. The only critical step is the separation between MYBs and MYB-like sequences. Emerging and species-specific MYB clades embedded in widely distributed clades are not a major concern, because it is possible to adjust the parameters of the analysis when analyzing isolated species e.g. members of the Lycophytes or Magnoliidae (see documentation for technical details). Only sequences at the basis of the MYB gene family tree could be at risk of being missed.

There are constrains that influence the size of the bait sequence collection. A narrower set of bait sequences could reduce the run time if a comprehensive investigation of numerous genome sequences of one taxonomic group is planned. An analysis with a more comprehensive set of MYB sequences could improve the quality of the results, but would also substantially increase the run time of the analysis. The generation of an ideal bait sequence set which represents the complete phylogenetic diversity of MYBs with a minimal number of sequences is a task for future studies.

Most steps are deterministic, but minor variations might occur as part of the tree building. However, no biologically relevant differences were observed during the analyses of 121 benchmarking datasets. Additionally, the results of analyses with BLAST-based selection of candidates were consistent with the results of corresponding analyses using HMMER for the identification of initial candidates.

## Conclusions

This approach only relies on standard tools which should be installed on most systems and are also easy to install if not available already. Technical checks on *A. thaliana* datasets indicate that the pipeline is accurately identifying MYBs. The performance allows the investigation of one species within minutes on ubiquitously available hardware. An investigation of the MYB gene families in *Castanea crenata* and *Croton tiglium* revealed expected patterns and demonstrated the potential to analyze genome and transcriptome sequence assemblies. While this approach is dedicated to the analysis of MYBs, it could be adjusted to investigate other transcription factor gene families.

## Availability and requirements

Project name: MYB_annotator.

 Project home page: https://github.com/bpucker/MYB_annotator

 Operating system(s): Linux.

 Programming language: Python3.

 Other requirements: dendropy, BLAST, HMMER, MAFFT, RAxML or FastTree2.

 License: GNU General Public License v3.0

 Any restrictions to use by non-academics: none.

## Supplementary Information

**Additional file 1.** MYB candidate classification example. A phylogenetic tree is analyzed to decide if a MYB candidate sequence falls into the in-group or into the out-group. This schematic illustration shows how the candidate sequences are classified.

**Additional file 2.** Assignment of MYB candidates to reference MYBs. This table contains the assignment of candidate MYBs to the reference MYBs and provides information about the functional annotation of the reference MYBs. The presented example shows the reference MYBs of *Arabidopsis thaliana* Col-0 and the candidate MYBs of *A. thaliana* Nd-1.

**Additional file 3.** data sets were screened for MYBs with the presented workflow. The number of sequences in the individual data sets and the run time of the analysis are given in the table.

**Additional file 4.** *Castanea crenata* MYBs. This FASTA file contains the coding sequences of the *C. crenata* MYBs that were identified in this study. 

**Additional file 5.** *Croton tiglium* MYBs. This FASTA file contains the coding sequences of the *C. tiglium* MYBs that were identified in this study. 

## Data Availability

The pipeline and the required input data sets are freely available via github: https://github.com/bpucker/MYB_annotator. Additionally, the released version was archived via zenodo (10.5281/zenodo.6174039). The analyzed transcriptomic and genomic resources are publicly available: *Castanea crenata* (BPMU01000001-BPMU01000781) and *Croton tiglium* (PRJNA416498).
