# Supplementary figures and images for: Automatic identification and annotation of MYB gene family members in plants
Source: BMC Genomics. 2022 Mar 19;23:220. doi: 10.1186/s12864-022-08452-5 (PMC8933966; doi:10.1186/s12864-022-08452-5)

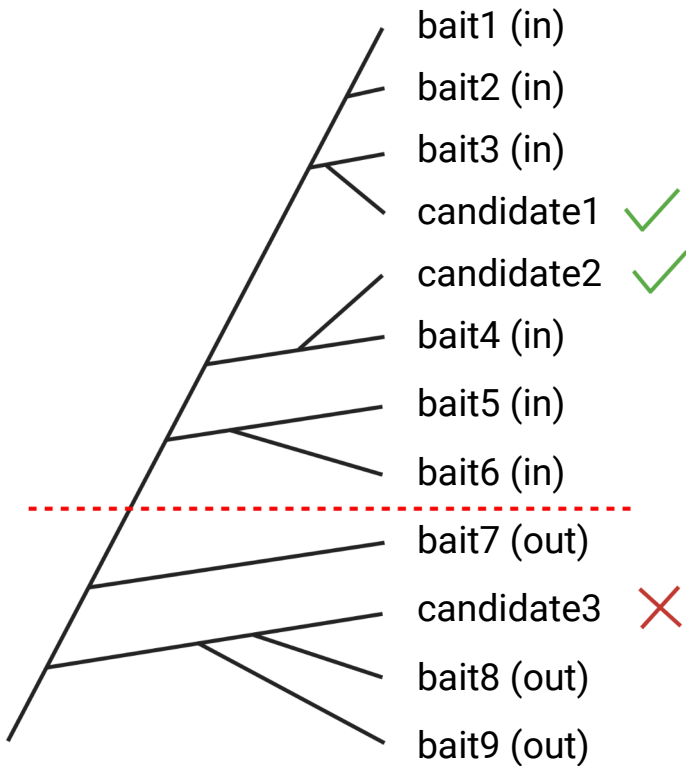

Supplement: Supplementary file 1 — Additional file 1. MYB candidate classification example. A phylogenetic tree is analyzed to decide if a MYB candidate sequence falls into the in-group or into the out-group. This schematic illustration shows how the candidate sequences are classified. [file 12864_2022_8452_MOESM1_ESM.pdf]
